# Lingual and hypoglossal nerve palsy following general anesthesia with laryngeal mask airway: a case report

**DOI:** 10.1186/s12871-026-03640-2

**Published:** 2026-01-21

**Authors:** Wenjun Yang, Limin Zhu, Meiyu Liu

**Affiliations:** ¹Department of Anesthesiology, Jiangdu People’s Hospital Affiliated to Yangzhou University, No. 100, Jiangzhou Road, Jiangdu District, Yangzhou, Jiangsu 225220 China

**Keywords:** Laryngeal mask airway, Hypoglossal nerve palsy, Lingual nerve palsy, Neuropraxia, Case report

## Abstract

**Background:**

Combined palsy of the hypoglossal and lingual nerves following laryngeal mask airway (LMA) use is a rare complication. This case report delineates the clinical course and management of this unique entity.

**Case presentation:**

A 71-year-old male developed left-sided tongue paralysis and sensory loss in the anterior two-thirds of the tongue 24 hours after hand surgery under LMA anesthesia. Central lesions were excluded via neuroimaging. Electromyography (EMG) confirmed a left hypoglossal nerve conduction delay, consistent with neuropraxia. A multimodal treatment regimen comprising a 5-day course of intravenous methylprednisolone followed by a 2-week oral prednisone taper, murine nerve growth factor for two weeks, and targeted rehabilitation was implemented. Complete neurological recovery was achieved within three months, as confirmed by clinical assessment and normalized electrophysiological studies.

**Conclusions:**

This case underscores that prolonged LMA use can lead to compound cranial neuropathy, likely due to mechanical compression at the tongue base. Early diagnosis, exclusion of central causes, and prompt initiation of combined anti-inflammatory, neurotrophic, and rehabilitative therapy are crucial for optimal recovery. Emphasis on individualized airway management and pressure monitoring in elderly patients is recommended to mitigate this risk.

## Background

The laryngeal mask airway (LMA) is a cornerstone of modern airway management in general anesthesia. While celebrated for its ease of use and reduced hemodynamic impact, it is not without risk. Rare complications include cranial neuropathies, with most reported cases involving the hypoglossal or recurrent laryngeal nerves in isolation or combination (Tapia’s syndrome) [1, 2]. However, the simultaneous injury of the hypoglossal nerve (CN XII, motor) and the lingual nerve (a branch of V3, sensory) is exceedingly rare. This combined injury represents a distinct clinical entity that is poorly characterized in the literature [3]. This case is unique for several reasons. First, it presents this rare compound neuropathy. Second, it occurs in an elderly patient following a prolonged (100-minute) surgical procedure, despite the use of a conventionally pressurized LMA. Furthermore, this report adds to the scientific literature by three aspects: (1) providing a detailed diagnostic workup that systematically rules out central and other peripheral causes; (2) outlining an effective, multimodal therapeutic regimen combining corticosteroids, neurotrophic factors, and early rehabilitation that resulted in complete functional recovery; (3) highlighting the critical importance of duration-of-use and patient age as key risk factors, thereby informing preventive strategies. This CARE-compliant case report aims to enhance clinical recognition and guide the management of this complex complication.

## Case presentation

### Patient information

A 71-year-old male (76 kg, American Society of Anesthesiologists [ASA] physical status II) was admitted for emergency debridement and repair of an open left-hand injury caused by mechanical trauma. The injury resulted from accidental entanglement with industrial machinery, causing multiple lacerations and partial extensor digitorum tendon transection. Importantly, this hand injury was etiologically unrelated to the subsequent cranial nerve palsy, as no head and neck neurovascular structures were involved.

His medical history included hypertension, well-controlled with amlodipine. Preoperative airway assessment revealed a Mallampati class I, with a three-fingerbreadth mouth opening and thyromental distance, showing no predictors of a difficult airway. Baseline neurological examination of the tongue was normal.

### Timeline

The key diagnostic and therapeutic events are summarized in Table 1.

**Table 1:** Timeline of clinical events from preoperative assessment to recovery

Time Point	Key Events
1 h preoperatively	Anesthesia evaluation completed, confirming no airway risk; informed consent for anesthesia obtained (including complication disclosure).
Intraoperative	Laryngeal mask airway (size 4, cuff pressure 20 cmH₂O) inserted; the patient was positioned in a supine position with the head maintained in a neutral alignment throughout the 100-minute procedure; general anesthesia maintained with stable vital signs.
24 h postoperatively	Patient reported “stiffness of the tongue during swallowing with food retention in the left oral cavity.” Physical examination revealed rightward tongue deviation upon protrusion.
48 h postoperatively	Methylprednisolone anti-inflammatory therapy initiated. Patient reported “slight improvement in dysphagia, but persistent numbness on the left tongue surface.”
3 days postoperatively	Electromyography (EMG) indicated abnormal conduction in the left hypoglossal nerve. Murine nerve growth factor therapy and swallowing rehabilitation training commenced.
3 weeks postoperatively	Patient reported “normal rice intake with improved tongue mobility.” Rightward tongue deviation upon protrusion was reduced.
6 weeks postoperatively	Sensory function in the anterior two-thirds of the tongue recovered. Patient reported “no numbness,” with left tongue muscle strength restored to Medical Research Council (MRC) grade IV.
3 months postoperatively	Tongue movement and sensation fully normalized. Repeat EMG showed no abnormalities, and the patient reported no discomfort.

### Diagnostic assessment

Clinical Findings: Examination revealed Medical Research Council (MRC) grade 4/5 strength in the left tongue. There was rightward tongue deviation upon protrusion and reduced pain sensation in the left anterior two-thirds of the tongue.

Electromyography (EMG) with a focus on nerve conduction studies (NCS) was performed on postoperative day 3. It revealed slowed conduction velocity (35 m/s) and prolonged latency in the left hypoglossal nerve, without denervation potentials—findings consistent with neuropraxia (a reversible conduction block). This early diagnosis guided immediate therapy.

Differential Diagnosis: Key differential diagnoses and reasons for their exclusion are presented in Table 2.

**Table 2:** Differential diagnoses considered and excluded

Differential Diagnosis	Basis for Exclusion
Central Tongue Palsy (e.g., Cerebral Infarction)	Absence of accompanying symptoms such as limb movement impairment or facial paralysis. No causative lesion detected on cranial magnetic resonance imaging (MRI).
Recurrent Laryngeal Nerve Injury	Fiberoptic laryngoscopy revealed normal vocal cord movement with no hoarseness (a hallmark manifestation of recurrent laryngeal nerve injury).
Iatrogenic Lingual Nerve Injury	The surgical procedure involved debridement of the left hand without intraoral manipulation, ruling out direct surgical trauma.

### Therapeutic intervention

A multifaceted treatment approach was employed:

1. Anti-inflammatory Therapy: Intravenous methylprednisolone (15 mg/day for 5 days) followed by a 2-week oral prednisone taper. Rationale: To reduce endoneurial edema and inflammatory mediator release associated with compressive neuropraxia [4].
2. Neurotrophic Support: Intramuscular murine nerve growth factor (30 µg/day for 2 weeks). Rationale: To promote axonal regeneration and support neural repair, particularly relevant in an elderly patient [5].
3. Rehabilitation: Initiated on postoperative day 3, including pharyngeal ice stimulation and tongue resistance exercises. Rationale: To prevent muscle atrophy, enhance neuromuscular re-education, and accelerate functional recovery of swallowing.

### Follow-up and outcomes

The patient’s recovery was systematically tracked. Tongue muscle strength improved from MRC grade 4/5 at onset to full grade 5/5 strength by 3 months. Sensory function normalized by 6 weeks. The mild tongue atrophy observed at 6 weeks had resolved by the 3-month follow-up. A repeat EMG confirmed normalization of the hypoglossal nerve conduction velocity (42 m/s). The patient resumed a normal diet and reported no residual symptoms, indicating a complete recovery.

## Discussion

**Fig. 1:**
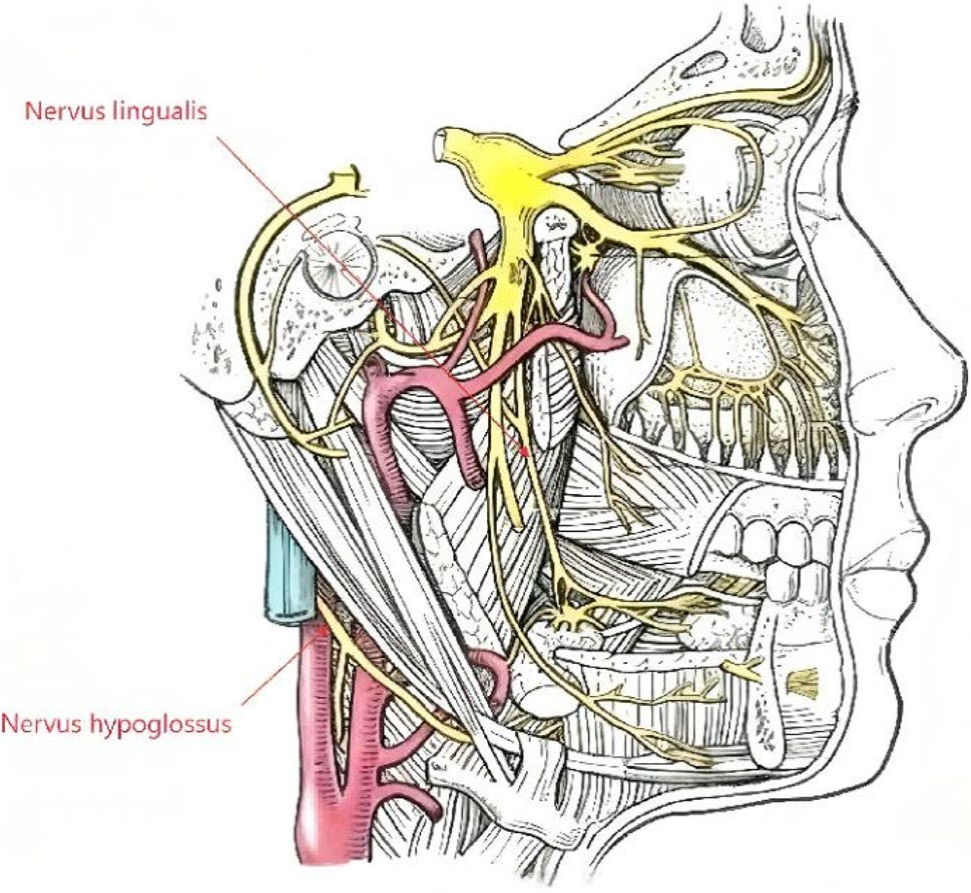
Anatomical course of the hypoglossal and lingual nerves. This schematic illustrates the path of the hypoglossal nerve (Nervus hypoglossus, CN XII) responsible for tongue movement, and the lingual nerve (Nervus lingualis, a branch of V3) providing sensation to the anterior two-thirds of the tongue. This figure was adapted and modified from “Trigeminal Nerve (Lateral View)” in Guo G, Wang X. Color Atlas of Human Anatomy. 3rd ed. Beijing: People’s Medical Publishing House; 2018. p. 283 [6]. The adaptation is published under the terms of the Creative Commons Attribution License

This case exemplifies a rare but significant complication of LMA use: combined motor-sensory neuropathy of the tongue. The co-occurrence of hypoglossal and lingual nerve palsies points to a shared mechanism of compression in the sublingual/oropharyngeal space, where both nerves are anatomically vulnerable to a deeply seated or over-pressurized LMA cuff [1]. As illustrated in Fig. 1, the hypoglossal nerve courses near the greater cornu of the hyoid bone while the lingual nerve runs laterally in the sublingual space—both potential compression sites from an improperly positioned LMA.

The patient’s advanced age was likely a predisposing factor. Age-related anatomical changes, such as reduced tissue compliance and superior positioning of the hyoid bone, may diminish the natural buffer against mechanical pressure [3, 7]. Additionally, the supine position with neutral head alignment during the 100-minute procedure, combined with intraoperative muscle relaxation, likely exacerbated sustained cuff pressure on the vulnerable hypoglossal and lingual nerves. This explains why nerve injury can occur even with LMAs with cuff pressures within the conventional range during prolonged procedures. The excellent recovery with conservative management aligns perfectly with neuropraxia—a reversible conduction block where axonal integrity is preserved, primarily characterized by localized myelin sheath injury without axonal disruption [4, 8].

### Treatment considerations

A multimodal regimen proves most effective for such compound nerve injuries. Corticosteroids address the inflammatory component and endoneurial edema [4], while neurotrophic factors support neural repair and regeneration—particularly relevant in elderly patients with diminished regenerative capacity. Early rehabilitation prevents muscle atrophy and promotes functional recovery. While this approach yields favorable outcomes, the recovery period for compound nerve injuries tends to be prolonged compared to isolated neuropathies.

### Prevention strategies

For prolonged procedures (> 90 min), especially in elderly patients, several preventive measures should be considered:

1. Use of a manometer to maintain cuff pressure ≤ 40 cmH₂O with regular intraoperative monitoring [5, 7].
2. Consideration of endotracheal intubation as an alternative airway management strategy.
3. Employment of fiberoptic guidance for optimal LMA positioning [8].
4. Preoperative anatomical assessment in high-risk patients.

### Limitations and future directions

This single case report, while informative, highlights the need for larger, multi-center studies to better characterize risk factors and establish evidence-based management guidelines. The absence of real-time intraoperative neuromonitoring and precise cuff pressure documentation represents a limitation in our understanding of the exact injury mechanism.

### Patient perspective

“The first day after surgery, my tongue felt stiff and numb, and I was worried it would never get better. After starting the medications and exercises, I improved every week. I’m very glad that my tongue is now back to normal and I can eat anything without any problem.”

## Conclusions

Combined hypoglossal and lingual nerve palsy is a rare, reversible complication of LMA anesthesia, primarily caused by mechanical compression. A high index of suspicion allows for prompt diagnosis. Early intervention with a combination of anti-inflammatory agents, neurotrophic factors, and physical rehabilitation can lead to excellent functional outcomes. Anesthesiologists should incorporate vigilant cuff pressure management and consider patient-specific anatomical factors to prevent this complication.

## Data Availability

All data underlying this report are included within the manuscript.
